# Risk factors associated with birth defects at a tertiary care center in Pakistan

**DOI:** 10.1186/1824-7288-38-68

**Published:** 2012-12-07

**Authors:** Mohammad Zeeshan Raza, Asfandyar Sheikh, Syed Salman Ahmed, Sajid Ali, Syed Mumtaz Ali Naqvi

**Affiliations:** 1 Dow Medical College, Dow University of Health Sciences, Karachi, Pakistan; 2 Baba-e-Urdu Road, Karachi, 74500, Pakistan

## Abstract

**Background:**

Birth defects are defined as those conditions that are substantially determined before or during birth and which are recognizable in early life. They are an important cause of morbidity and mortality in infants. The main objective of the study was to determine the association of certain risk factors with birth defects occurring in pediatric patients seeking care in Civil Hospital, Karachi.

**Methods:**

This was a prospective, cross-sectional study conducted at Department of Pediatrics: Units I, II and III of Civil Hospital Karachi, which is a tertiary care hospital located in the city center. These units provide care to pediatric patients from all over the country, with majority belonging to a low socioeconomic background. All infants with at least one birth defect were approached and their mothers interviewed. Demographics of both the mother and the infant were noted. Questions regarding possible exposure to risk factors were asked. Infants who were not accompanied by their mothers were excluded from the study.

**Results:**

A total of 587 out of 669 infants completed the study successfully. Of these, defects related to urogenital system (19.9%) were the commonest, followed by those related to eye (16.9%), musculoskeletal system (12.9%), body wall defects (12.3%), oral cavity (12.1%), central nervous system (10.9%), gastrointestinal tract (3.2%), cardiovascular system (2.7%) and those related to ear, nose and throat (1.2%).

**Conclusion:**

669(4.1%) out of a total of 16,394 pediatric patients visiting the hospital during our study were identified as having at least one birth defect. The commonest ones were those related to the eye and the urogenital system. The main factors that influence the occurrence can be grouped into maternal, socioeconomic, nutritional and educational.

## Introduction

Birth defects are defined as ‘those conditions that are substantially determined before or during birth and which are, in principle, recognizable in early life’ [1]. Some of these defects are classified as major and may require surgical intervention and/or cause death of the infant. Others are classified as minor, which are significantly detrimental to the quality of life and health of the patient. However, this classification is somewhat ambiguous, as some minor anomalies can be associated with underlying major defects. This association could be 3% in patients having one, 10% in patients having two, and 20% in patients having more than three anomalies [2].

Thus, a broad definition of birth defects includes not only anatomical defects but also molecular and cellular abnormalities present at birth [3]. A WHO document in 1972, however, maintained that the term “congenital malformations” should be confined to structural defects at birth, with the term “congenital anomaly” being used to include all biochemical, structural and functional disorders present at birth [1]. The word “birth defects” has therefore been used throughout the study in order to avoid ambiguity.

Birth defects usually occur during organogenesis (between 3rd and 8th week of gestation). They may result in complete or partial absence of an anatomical part or alteration of its normal configurations. Mostly, these are caused by environmental or genetic factors acting independently or in concert. Major structural anomalies occur in 2% to 3% of live born infants. An additional 2% to 3% are recognized in children by age 5 years, for a total of 4% to 6% [2].

Some of these disorders are obvious at birth, for e.g. cleft palate; some, such as congenital dislocation of the hip (CHD), may escape early detection, while others may not become apparent until much later in life, for e.g. patent ductus arteriosus (PDA). Internal defects, when they are not lethal, may often go unrecognized. However, in any case, these defects are a major cause of morbidity and mortality in infants worldwide, accounting for as many as 260,000 deaths (7% of all neonatal deaths) in the year 2004 alone [4].

Many studies have been conducted to determine the association of various risk factors with the incidence of birth defects. For example, folic acid supplementation has long been implicated in the prevention of neural tube defects and other major defects [5]. Similarly, maternal cigarette smoking has been associated with an increased risk of cardiovascular and other malformations [6]. Consanguineous marriage, which is a common practice in the country, has also been found out to have a role [7,8].

The prevalence of birth defects varies widely, depending on the geographical locale. It was found to be 2.07% in Turkey, 2.39% in Europe, 1.5% in Japan and 1.96% in the United States [9-12]. These numbers, just as in our case, are based on studies conducted in limited settings, such as hospitals. These do not take into account all those infants who are born at other centers. Their prevalence may be very different from those reported for tertiary care centers, as they are often visited by patients belonging to a wide range of socioeconomic statuses, which has been implicated as a possible risk factor for birth defects [13].

The aim of our study was to assess the risk factors associated with various birth defects in pediatric patients seeking care in Civil Hospital, Karachi.

## Materials and Methods

### Study setting and participants

This was a prospective cross sectional, interview-based study conducted during a three year period from 20th February, 2009 to 15th March, 2012 at Pediatrics I, II and III wards in Civil Hospital, Karachi, which is a public sector, tertiary care hospital. This hospital is a host to visitors from all parts of the country.

Information regarding patients was obtained from patient records at the beginning of each session of study. Those found to have at least one birth defect were approached and their attendants (mothers) were interviewed. Those, whose mothers were not available at the time of interview, were checked at the end of the next session for a maximum of 5 times. The type of defect was noted down from the patient files along with other parts of the questionnaire.

### Inclusion and Exclusion Criteria

All infants who were diagnosed with having at least one birth defect were included. Those subjects, whose mothers were not available for interview after 5 attempts, were excluded from the study.

### Ethics

The Ethical Review Board of Dow University of Health Sciences approved the study. Subjects’ attendants were informed of their right to refuse and of the respect of the confidentiality of their answers. Informed, written consent was obtained from all attendants.

### Questionnaire

The questionnaire was divided into four sections. The first section dealt with demographic data of the child, which consisted of age, sex, birth weight, ethnicity, religion, residence and socioeconomic status. The second section was concerned with data regarding the child’s mother. It included questions regarding maternal comorbidities, gestational age, gestational period, occupation and education level of the mother. The third section contained a series of Yes/No questions regarding exposure to various risk factors. The fourth section involved noting down the name of the specific defect from the patient file.

A pretest of the interview questionnaire was conducted on a sample of 13 infants to determine its effectiveness. The questionnaire was edited in order to overcome the shortcomings encountered in the pretest.

### Analysis of Data

Data from the questionnaire was entered in SPSS (Statistical Package for the Social Sciences) version 14 for analysis.

## Results

A total of 16,394 pediatric patients visited the hospital during our study period out of which 669(4.1%) were classified as having at least one birth defect. A total of 587 patients out of 669 were evaluated completely during the course of study (response rate 87.7%). The decrease was due to non-availability of child’s mother, or due to failure to give consent.

### Patients’ Demographics

Data regarding patients’ demographics is illustrated in Table 1. Mean ± SD birth weight was 3139 ± 242 grams. Mean ± SD household income was Rs. 9314 ± 557 (~USD 100 ± 6).

**Table 1:** Patients' demographics

		**N(%)**
Gender	Male	401(68.3)
	Female	186(31.7)
Birth Weight	<2500	162(27.6)
	2500-4000	279(47.5)
	>4000	146(24.9)
Ethnicity	Sindhi	227(38.7)
	Punjabi	34(5.8)
	Pathan	131(22.3)
	Balochi	68(11.6)
	Other	31(5.3)
	Multiethnic	96(16.4)
Religion	Islam	496(84.5)
	Hinduism	69(11.8)
	Christianity	15(2.5)
	Other	7(1.2)
Residence	City	253(43.1)
	Village	334(56.9)
Income	<5000	342(58.3)
	5000-20000	186(31.7)
	>20000	59(10.0)

### Maternal Characteristics

Maternal Characteristics are elaborated in Table 2. Mean ± SD age of the mothers at pregnancy was 29.5 ± 3.3 years with the minimum being 17 years and maximum being 46 years.

**Table 2:** Maternal characteristics

		**N(%)**
Gestational Age	<20	39(6.6)
	20-25	87(14.8)
	26-30	173(29.5)
	31-35	227(38.7)
	>40	61(10.4)
Maternal Occupation	Housewife	379(63.9)
	Labor	143(24.4)
	Skilled Job	62(10.6)
	Business	3(0.5)
Maternal Education	None	384(65.4)
	Could Write Own Name	107(18.2)
	Undergraduate	91(15.5)
	Graduate	5(0.9)
Maternal Comorbids	Diabetes Mellitus	14(2.4)
	Hypertension	81(13.3)
Pregnancy Period	Preterm	227(38.7)
	Term	294(50.1)
	Postterm	66(11.2)

### Risk Factors

Table 3 shows the percentage of mothers exposed to different risk factors.

**Table 3:** Risk factors

	**Yes**	**No**
	**N(%)**	**N(%)**
Maternal Folate Supplementation	214(36.5)	373(63.5)
Maternal Cigarette Smoking	106(18.1)	481(81.9)
Other Addictions (Maternal)	59(10.1)	528(89.9)
Maternal X-rays Exposure	17(2.9)	570(97.1)
Family History	114(19.4)	473(80.6)
Consanguineous Marriage	227(38.7)	360(61.3)
Trauma During Pregnancy	25(4.3)	562(95.7)
Landfills/Industries Near Residence	142(24.2)	445(75.8)

### Birth Defects

**Figure 1:**
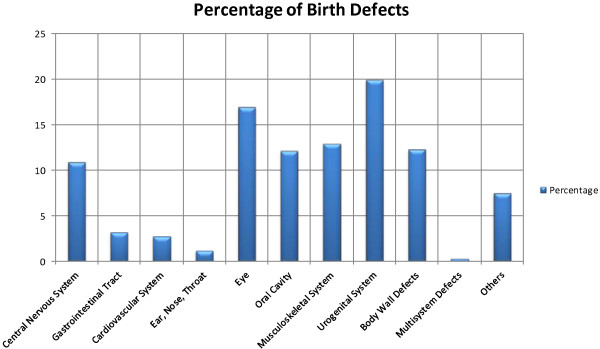
Percentage of Birth Defects.

Table 4 provides the percentages of different birth defects, grouped according to organ systems. Figure 1 is a graphical representation of the data. Table 5 provides an in depth summary of different defects. Male : Female ratio was only calculated for those defects, where at least one of either was present.

**Table 4:** Malformations grouped by Regions

	**N(%)**
Central Nervous System	64(10.9)
Gastrointestinal Tract	19(3.2)
Cardiovascular System	16(2.7)
Ear, Nose, Throat	7(1.2)
Eye	99(16.9)
Oral Cavity	71(12.1)
Musculoskeletal System	76(12.9)
Urogenital System	117(19.9)
Body Wall Defects	72(12.3)
Multisystem Defects	2(0.3)
Others	44(7.5)

**Table 5:** Summary of congenital malformations

		**Preterm**		**Term**		**Post term**		
		**Male**	**Female**	**Male**	**Female**	**Male**	**Female**	**Male:Female ratio**
		**N**	**N**	**N**	**N**	**N**	**N**	
CNS	Hydrocephalus	13	7	16	4	1	2	1:0.43
	Neural Tube Defects	7	4	1	1	0	3	1:1
	Congenital Schwannoma	0	0	2	0	0	0	NC
	Congenital Cranipharyngioma	1	0	2	0	0	0	NC
GI	Small Intestine Obstruction	1	1	0	0	0	0	1:1
	Hirschprung Disease	0	0	1	0	0	1	1:1
	Ectopic Anus	0	0	1	0	0	0	NC
	Imperforate Anus	4	3	2	3	1	1	1:1
CVS	Patent Ductus Arteriosus	6	3	1	0	0	0	1:0.43
	Ventricular Septal Defect	0	2	2	0	1	0	1:0.67
	Atrial Septal Defect	1	0	0	0	0	0	NC
ENT	Laryngeocele	0	0	1	0	0	0	NC
	Tracheoesophageal Fistula	0	0	1	0	0	0	NC
	Fistula in Neck	1	0	0	0	0	0	NC
	Congenital Torticollis	1	0	0	0	1	0	NC
	Choanal Atresia	0	1	0	1	0	0	NC
Eye	Aphakia	0	2	1	0	0	0	1:2
	Retinoblastoma	36	11	22	11	0	0	1:0.38
	Congenital Strabismus	2	2	2	2	0	0	1:1
	Glaucoma	0	0	1	1	0	0	1:1
	Cataract	4	1	1	0	0	0	1:0.2
Oral Cavity	Ankyloglossia	0	0	4	4	1	2	1:1.2
	Palatal Fistula	1	0	2	0	0	0	NC
	Cleft Lip Alone	6	4	5	3	1	1	1:0.67
	Cleft Palate Alone	1	3	1	4	1	2	1:3
	Cleft Lip+Palate	7	5	6	4	1	2	1:0.79
Musculo-skeletal System	Extra Digit	0	0	4	1	0	0	1:0.25
	Syndactyly	0	0	1	2	2	0	1:0.67
	Talipes Equinovarus	12	3	25	15	3	7	1:0.63
	Ankylosed Hip Joint	1	0	0	0	0	0	NC
Urogenital System	Urethral Fistula	3	0	2	1	1	0	1:0.17
	Meatal Stenosis	1	0	4	0	0	0	NC
	Penile Torniquet	1	0	2	0	0	0	NC
	Congenital Hydrocele	5	4	19	6	7	1	1:0.35
	Congenital Cryptorchidism	15	0	6	0	1	0	NC
	Ambiguous Genitalia	0	1	0	0	0	0	NC
	Congenital Ovarian Cyst	0	0	0	0	0	1	NC
	Hypospadias	14	0	19	0	3	0	NC
Body Wall Defects	Supraumbilical Hernia	0	1	0	1	0	0	NC
	Umbilical Hernia	0	0	2	1	0	0	1:0.5
	Umbilical Polyp	1	0	0	0	0	0	NC
	Umbilical Sinus	0	0	1	0	0	0	NC
	Epigastric Hernia	1	1	0	0	0	0	1:1
	Inguinal Hernia	7	5	22	10	12	7	1:0.54
Multisystem Defects	Anorectal+Meatal Stenosis	0	0	0	1	0	0	NC
	Anoscrotal Malformation	0	0	1	0	0	0	NC
Others	Labial Hemangioma	0	0	2	1	0	0	1:0.5
	Lipodermoid Cyst	0	0	1	2	0	0	1:2
	Congenital Epidermoid Cyst	0	0	5	1	0	0	1:0.2
	Xeroderma Pigmentosum	0	0	4	3	0	0	1:0.75
	Congenital Angiofibroma	0	0	2	1	0	0	1:0.5
	Cystic Hygroma	0	0	2	4	0	0	1:2
	Cystic Adenoid	0	0	1	1	1	0	1:0.5
	Cystic Nodule	0	0	2	1	0	0	1:0.5
	Congenital Dermoid Cyst	0	0	2	1	0	0	1:0.5
	Bifid Nose	0	0	2	0	0	0	NC
	Multiple Malformations	3	1	1	0	0	0	1:0.25

## Discussion

This is only the first study of its kind in Pakistan that aims to assess the risk factors for certain birth defects at a tertiary care center. It is also, according to our knowledge, the first prospective, interview-based study conducted on this topic. The percentage of affected individuals in our study (4.1%) is also quite high compared to studies from other parts of the world. This could be due to the fact that the study was conducted in a hospital setting, where only those infants are admitted who need special care.

A higher percentage of males was found to be affected compared to females. This finding is consistent with that of Shaw et al. who observed an increased risk for most systems even after adjusting for confounders [14]. The male : female ratio for most systems in our study also favored the males, as can be seen from Table 5. Socioeconomic status for most was poor, with a mean salary of Rs. 9314 (USD 100). This could be one of the reasons for a high percentage of affected patients in our setting, as socioeconomic status is an important risk factor for birth defects [13,15,16].

A large percentage of our subjects (38.7%) was born before 37 weeks. Kase et al. reported a reciprocal relationship between being born preterm and the presence of birth defects, which is consistent with our findings [17]. However, the mean weight suggests that most infants had a normal birth weight. This is surprising as most studies report a higher risk of birth defects in infants with low birth weight [18,19].

Maternal factors have been found to play an important role in the presentation of birth defects. Most mothers, in our study group, were over the age of 30. A high incidence of defects has been observed for both extremes of ages in multiple studies [20-23]. Maternal occupation has also been implicated in the incidence of birth defects [24-26]. However, in our study, majority of mothers were housewives. This could be partly explained by the fact that, in backward areas of Pakistan, women are not allowed to work outside the confines of their homes.

Maternal Education also has an indirect effect on the incidence of birth defects [27]. In our study, 83.6% mothers were uneducated (including 18.2% who could just write their names). Awareness regarding periconceptual supplementation with folate, and abstention from certain drugs plays a major role in the incidence.

Certain maternal comorbidities have been linked with an increased incidence of birth defects. Mothers with preexisting or gestational diabetes generally have a higher incidence of birth defects, with the cardiovascular, musculoskeletal and central nervous systems being the most affected [28-30]. However, in our study, a significant proportion did not have diabetes at any stage of pregnancy.

Among maternal risk factors, folate supplementation, cigarette smoking and exposure to x-rays occupy a significant position. Neural tube defects, which include spina bifida and encephaloceles, have long been linked to folic acid supplementation [31-33]. In our study, a considerable majority (63.5%) did not receive periconceptual folate. This percentage increased to 81.3% in mothers of infants with neural tube defects. This can be largely attributed to the low educational level of majority of the participants’ mothers, which is directly related to awareness regarding folate supplementation [34]. Maternal smoking is also a moderate risk factor for certain malformations, especially congenital heart defects [35-37]. A potential confounder for this could be the fact that infants born to smoking mothers are largely preterm, or have a low birth weight [38-41]. In our sample, only 18.1% of the mothers had smoked at least once during their pregnancy. Exposure to x-rays and other radiations has also been implicated in increasing the risk for birth defects [42].

Among social factors, consanguineous marriage has been repeatedly found to have an association with birth defects [43,44]. In our study, 38.7% were married to either first or second cousins. Similarly, a positive family history has also been found to be associated with an increase in risk [45]. Presence of industries and landfills has also been found to play an important role [46,47].

## Conclusion

In conclusion, the percentage of affected individuals in our setting has been found to be greater than in other similar studies. The main factors that influence the incidence can be grouped into maternal, socioeconomic, nutritional and educational.

### Limitations

The most important limitation of our study was that we included only live infants, who had their mother available to answer the questions. Secondly, stillbirths were not included in the study. The study was conducted in a hospital setting which does not truly represent the percentage throughout the country.

This is one of the first studies on the epidemiology of congenital malformations in Pakistan. In our effort to make it as extensive as we possibly could, we included even those malformations, whose classification as congenital was debatable, or which although predominantly found after birth, also occurred as birth defects.

Certain malformations, such as congenital heart defects, have been underrepresented in our study. Their low percentage finds its roots in the prospective nature of the study. Although all attempts were made to ensure the maximum number of cases were included, a certain percentage could not be reached and thus not included in the study. Furthermore, although such malformations affect a significant percentage of Pakistani infants, a large percentage of them either dies, or is referred to special centers (eg NICVD) for emergency treatment, with only few remaining in tertiary care centers. Since our study was conducted in just one tertiary care center, many cases could not be included. This has led to a significantly low percentage of these relatively common birth defects in our sample.

## Competing interests

The authors declare that they have no conflict of interests.

## Authors’ contributions

AS, MZR and SSA conceived the idea and were involved in data collection, analysis and drafting of the manuscript. SA and SMAN were involved in data collection and critically revising the manuscript. All authors have read and approved the manuscript.
